# Effects of *Ixeris dentata* water extract and caffeic acid on allergic inflammation *in vivo* and *in vitro*

**DOI:** 10.1186/s12906-015-0700-x

**Published:** 2015-06-24

**Authors:** Yong-Deok Jeon, Ji-Ye Kee, Dae-Seung Kim, Yo-Han Han, Sung-Hoon Kim, Su-Jin Kim, Jae-Young Um, Seung-Heon Hong

**Affiliations:** Department of Oriental Pharmacy, College of Pharmacy, Wonkwang-Oriental Medicines Research Institute, Wonkwang University, Jeonbuk, 570-749 Republic of Korea; Department of Oriental Medicine Resources, College of Environmental & Bioresources Sciences, Chonbuk National University, Iksan, Republic of Korea; College of Korean Medicine, Institute of Korean Medicine, Kyung Hee University, 26 Kyungheedae-ro, Dongdaemun-gu, Seoul Republic of Korea; Department of Cosmeceutical Science, Daegu Hanny University, Yugok-dong, Kyungsan, 712-715 Republic of Korea

**Keywords:** *Ixeris dentata* Nakai, Caffeic acid, Allergic inflammation, Keratinocytes, Mast cells, Mitogen-activated protein kinases

## Abstract

**Background:**

*Ixeris dentata* Nakai has been used for the treatment of mithridatism, calculous, indigestion, pneumonia, hepatitis, and tumors in Korea, China, and Japan. However, the effect of a water extract of *Ixeris dentata* (ID) and its molecular mechanism on allergic inflammation has not been elucidated. In this study, we attempted to evaluate the effects of ID and its major compound caffeic acid on allergic inflammation *in vivo* and *in vitro*.

**Methods:**

ID was applied to 2, 4-dinitrofluorobenzene (DNFB)-induced atopic dermatitis (AD)-like skin lesion mice and immune cell infiltration, cytokine production, and the activation of mitogen-activated protein kinases (MAPKs) were investigated*.* Moreover, the effect of ID on compound 48/80-induced anaphylactic shock was investigated in a mouse model. The human keratinocyte cell line (HaCaT cells) and human mast cells (HMC-1) were treated with ID or caffeic acid to investigate the effects on the production of chemokines and proinflammatory cytokines and on the activation of MAPKs.

**Results:**

ID inhibited the serum levels of IgE and interleukin (IL)-1β in DNFB-induced AD-like skin lesion mouse models and suppressed anaphylactic shock in the mouse models. ID and caffeic acid inhibited the production of chemokines and adhesion molecules in HaCaT cells. In addition, ID reduced the release of tumor necrosis factor-α and IL-8 via the inhibition of MAPKs phosphorylation in HMC-1 cells.

**Conclusions:**

These results suggest that ID is a potential therapeutic agent for allergic inflammatory diseases, including dermatitis.

## Background

Atopic dermatitis (AD), also known as atopic eczema, is a common, chronic and allergic inflammatory skin disease [1]. The worldwide prevalence rate of AD is nearly 10-20 % in children and 1–3 % in adults. Genetic and environmental factors are important causes in many AD patients [2]. Immune system disorders cause AD, which is related to several types of cells (T lymphocytes, macrophages, mast cells, and keratinocytes), specifically T helper (Th) cell dysfunction and IgE production [3, 4].

*Ixeris dentata* Nakai (Compositae) has been used for the treatment of mithridatism, calculous, indigestion, pneumonia, hepatitis, and tumors in Korea, China, and Japan [5]. It has been reported that *Ixeris dentata* has neuroprotective effects [6], anti-diabetic effects [7], protection effects against colitis and skin inflammation [8, 9], and anti-allergic effects [10, 11]. In this study, the effect of the *Ixeris dentata* water extract (ID) on allergic inflammatory reactions via MAPKs signaling was investigated in human keratinocytes and human mast cells and was also assessed *in vivo.*

Keratinocytes, which are the main type of epidermal cells, play a key role in the pathogenesis of AD [12]. Epidermal keratinocytes release various inflammatory mediators, such as chemokines and adhesion molecules by tumor necrosis factor (TNF)-α and interferon (IFN)-γ stimulations [13, 14]; these factors are involved in the development of inflammatory skin diseases, including AD. Thymus and activation-regulated chemokine (TARC or CCL17) as well as macrophage-derived chemokine (MDC or CCL22), which are Th2 chemokines, mediate the migration of lymphocytes to inflammatory sites and aggravate AD [15]. Additionally, the levels of TARC and MDC are elevated in serum and skin lesions of AD patients [16, 17].

Mast cells are hematopoietic cells that originate from progenitor cells in the bone marrow. They are considered as key effector cells in IgE-mediated immediate hypersensitivity and allergic disorders [18]. In response to various stimuli, mast cells secrete a variety of bioactive substances, such as histamine and several proinflammatory cytokines, including interleukin (IL)-8 and TNF. These mediators contribute to inflammation through the recruitment and activation of immune cells [19]. Mast cells are increased in a majority of AD patients and are believed to be involved in the pathogenesis of AD [20].

Nuclear factor-kappaB (NF-κB) plays a pivotal role in the regulation of immune and inflammatory responses through the expression of cytokine, chemokine, and adhesion molecules. When IκB proteins are phosphorylated and degraded after stimulation, NF-κB is translocated into the nucleus and initiates the transcription of genes that are crucial in the development of inflammatory diseases. Therefore, the activation of NF-κB is important in the progression of allergic inflammation [21–23].

Mitogen-activated protein kinases (MAPKs) signaling pathway controls a vast array of physiological processes. In multicellular organisms, there are three well-characterized subfamilies of MAPKs. These MAPKs include the extracellular signal-regulated kinases (ERKs); the c-jun N-terminal kinases (JNKs); and the p38 MAPKs [24]. It has shown to be important in the proliferation, activation, degranulation, and migration of various immune cells. The activation of MAPKs is associated with the allergic inflammatory response via the translocation of NF-κB, which causes the production of proinflammatory cytokines and chemokines [25–27]. Synthetically, the inhibition of NF-κB and MAPKs activation has been suggested as an anti-inflammatory strategy in allergic inflammation.

In this study, we evaluated the effect of ID on DNFB-induced AD-like skin lesion and anaphylactic shock in mouse models. We also examined the inhibitory effects of ID and its major compound, caffeic acid, on the productions of TNF-α/IFN-γ-induced chemokines and adhesion molecules in the human keratinocyte HaCaT cell line. Moreover, the effects of ID and caffeic acid on phorbol 12-myristate 13-acetate (PMA)/calcium ionophore A23187 (A23287)-induced proinflammatory cytokines productions as well as its mechanisms were investigated in human mast cells (HMC-1).

## Methods

### Antibodies and reagents

2, 4-dinitrofluorobenzene (DNFB), compound 48/80, 3-(4, 5-Dimethylthiazol-2-yl)-2, 5-diphenyltetrazolium bromide (MTT), PMA, and A23187 were purchased from Sigma-Aldrich Chemical Co. (St. Louis, MO, USA). Avidin peroxidase (AP), fetal bovine serum (FBS), Roswell Park Memorial Institute (RPMI) 1640, and Iscove's Modified Dulbecco's Medium (IMDM) were purchased from Gibco BRL (Grand Island, NY, USA). Anti-human TNF-α/IL-6/IL-8, recombinant TNF-α/IL-6/IL-8, biotinylated TNF-α/IL-6/IL-8, anti-mouse IgE/IL-1β, recombinant IgE, IL-1β, biotinylated IgE, and IL-1β were purchased from BD Pharmingen (San Diego, CA, USA). Anti-phospho p38 antibody was purchased from Cell Signaling Technology, Inc. (Danvers, MA, USA). Anti-phospho ERK, JNK, IκBα, anti-p38, JNK, NF-κB, histone, and GAPDH antibodies were purchased from Santa Cruz Biotechnology, Inc. (Santa Cruz, CA, USA). Anti-ERK, and α-tubulin antibodies were purchased from Bioworld Technology (Minneapolis, MN, USA).

### Animals

BALB/c mice (5 weeks, male, 19–20 g) were purchased from Da-Mool science (Daejeon, Republic of Korea). The animals were housed in a laminar air-flow room maintained at a temperature of 22 ± 1 °C and a reactive humidity level of 55 ± 1 % throughout the study. All animal experimental processes were approved by the Wonkwang University Institutional Animal Care and Use Committee (Confirmation No. WKU14-34).

### Preparation of ID

*Ixeris dentata* was obtained from the Wonkwang Institute of Biomedical Engineering Research and authenticated by Professor S. J. Park, College of Korean Medicine, Wonkwang University. A voucher specimen (No. 05-17-12) was deposited at the Herbarium of the College of Pharmacy, Wonkwang University. It was prepared by decocting a dried prescription of herbs with distilled water for 3 h. The extract was filtered, lyophilized, and kept at 4 °C. The samples were dissolved in distilled water and then filtered through a 0.22 μm syringe filter. The yield of the dried extract from the starting materials was about 7.8 %. A caffeic acid content analysis revealed a concentration of 4 ± 0.00 mg/g (0.4 ± 0.00 %) [8].

### DNFB-induced dermatitis

Experiments were performed according to a previously described method [28]. The dorsal skin of BALB/c mice (n = 7) was shaved by depilatory equipment before the experiment. The mice were sensitized with 100 μl of 0.15 % DNFB in an acetone olive oil (3: 1) or a vehicle applied to the dorsal skin twice each week for six weeks. ID (1 mg/kg) was orally administrated six weeks during the experiment.

### Histological analysis

Dermatitis skins samples were excised from the mice 24 h after the final DNFB application and fixed with 10 % formaldehyde, embedded in paraffin wax, routinely processed and then sectioned into 4-μm-thick slices. The skin sections were then stained with hematoxylin and eosin and examined by light microscopy for the presence of edema and for inflammatory cell accumulation.

### Enzyme-linked immunosorbent assay (ELISA)

96-well plates were coated with capture antibodies overnight at 4 °C. After washing with PBST, samples and cytokine standards were added and incubated at 37 °C for 2 h. The plates were washed and biotinylated antibodies were added. The samples were then left for 2 h. After washing, the plates were incubated with AP for 30 min in an incubator set to a temperature of 37 °C. Finally, TBS substrate solution was added and color development was measured by a microplate reader at 405 nm.

### Preparation of total cell lysates and nuclear extracts and western blot analysis

Stimulated cells were rinsed with ice-cold PBS and lysed using lysis buffer (iNtRon Biotech, Seoul, Republic of Korea) for 1 h. Total cell lysates were centrifuged for 10 min at 4 °C, after which the supernatants were obtained. Nuclear extracts were prepared using NE-PER Nuclear and Cytoplasmic Extraction Reagents (Pierce Thermo Scientific, Rockford, USA). After bicinchoninic acid (BCA) protein quantification, the supernatant was mixed with a 2× sample buffer, boiled for 5 min, separated by 10 % SDS-polyacrylamide gel electrophoresis, and then transferred to nitrocellulose membranes. The membranes were blocked with 10 % skim milk for 1 h and 30 min and incubated for 3 h with primary antibodies. They were then incubated with secondary antibodies for 45 min. Protein bands were detected using an ECL solution.

### Anaphylactic shock

The mice (n = 5) were administrated intraperitoneal injections of compound 48/80 (8 mg/kg). Control mice received a vehicle (PBS) and ID (0.01 - 1 g/kg) was orally administrated 1 h prior to the injection of compound 48/80. Mortality was monitored for 1 h after the induction of anaphylactic shock.

### Cell culture

Human immortalized keratinocytes (HaCaT cells) in RPMI 1640 supplemented with 10 % FBS, 100 units/ml of penicillin, and 100 μg/ml of streptomycin were cultured in a humidified atmosphere of 5 % CO_2_/95 % air at 37 °C. The human mast cell line (HMC-1) were cultured in IMDM supplemented with 10 % FBS, 100 units/ml of penicillin, and 100 μg/ml of streptomycin in a humidified atmosphere of 5 % CO_2_/95 % air at 37 °C.

### Cell viability

An MTT assay was performed to investigate the cell toxicity of ID and caffeic acid. MTT solution (500 μg/ml) was added, followed by incubation at 37 °C for 4 h. The crystallized formazan was dissolved in DMSO and the absorbance was measured at 540 nm by a microplate reader.

### Reverse-transcriptase polymerase chain reaction (RT-PCR)

Total RNA was isolated from HaCaT cells according to the manufacturer's specifications using an easy-BLUE RNA extraction kit (INtRON Biotech., Seoul, Republic of Korea). Total RNA and oligo(dT)_15_ were heated to 75 °C for 5 min and then placed on ice for at least 1 min. Each sample was reverse-transcribed to cDNA for 60 min at 42 °C using a cDNA synthesis kit. RT-PCR was performed with 1 μl of a cDNA mixture with a final volume of 20 μl with 10 pM of cytokine primers, i-MAX^TM^ ІІ DNA Polymerase (5 U/μl), a 10× PCR buffer (300 mM Tris–HCl (pH 9.0); 300 mM salts containing K^+^ and NH_4_^+^, with 20 mM Mg^2+^), a dNTP mixture (2.5 mM/each), DEPC (diethylpyrocarbonate)-treated water. Amplification was conducted for 35 cycles, as follows: denaturation at 94 °C for 30 s, annealing at 60 °C for 30 s, and extension at 72 °C for 45 s. Products were separated by electrophoresis on a 1.5 % agarose gel and visualized by staining with ethidium bromide. The gels were certificated using a CN-TFX device (Vilber Lourmat, Marne-la-Vallée, France). The primer sequences are shown in Table 1.

**Table 1:** Sequences of the RT-PCR primers

Genes	Forward (5’-3’)	Reverse (5’-3’)
IL-8	GTCCTTGTTCCACTGTGCCT	GCTTCCACATGTCCTCACAA
TARC	ACTGCTCCAGGGATGCCATCGTTTTT	ACAAGGGGATGGGATCTCCCTCACTG
MDC	AGGACAGAGCATGGCTCGCCTACAGA	TAATGGCAGGGAGGTAGGGCTCCTGA
ICAM-1	CACCCTAGAGCCAAGGTGAC	CATTGGAGTCTGCTGGGAAT
MMP-9	CACTGTCCACCCCTCAGAGC	GCCACTTGTCGGCGATAAGG
β-actin	GGACTTCGAGCAAGAGATGG	AGCACTGTGTTGGCGTACAG

### Statistical analysis

The results are shown as a summary of the data from three experiments and are presented as the mean ± SD. A statistical evaluation of the results was performed by the Student’s *t*-test. For all experiments, *p <* 0.05 was considered to be significant.

## Results

### Effect of ID on DNFB-induced dermatitis and serum IgE and IL-1β levels

**Fig. 1:**
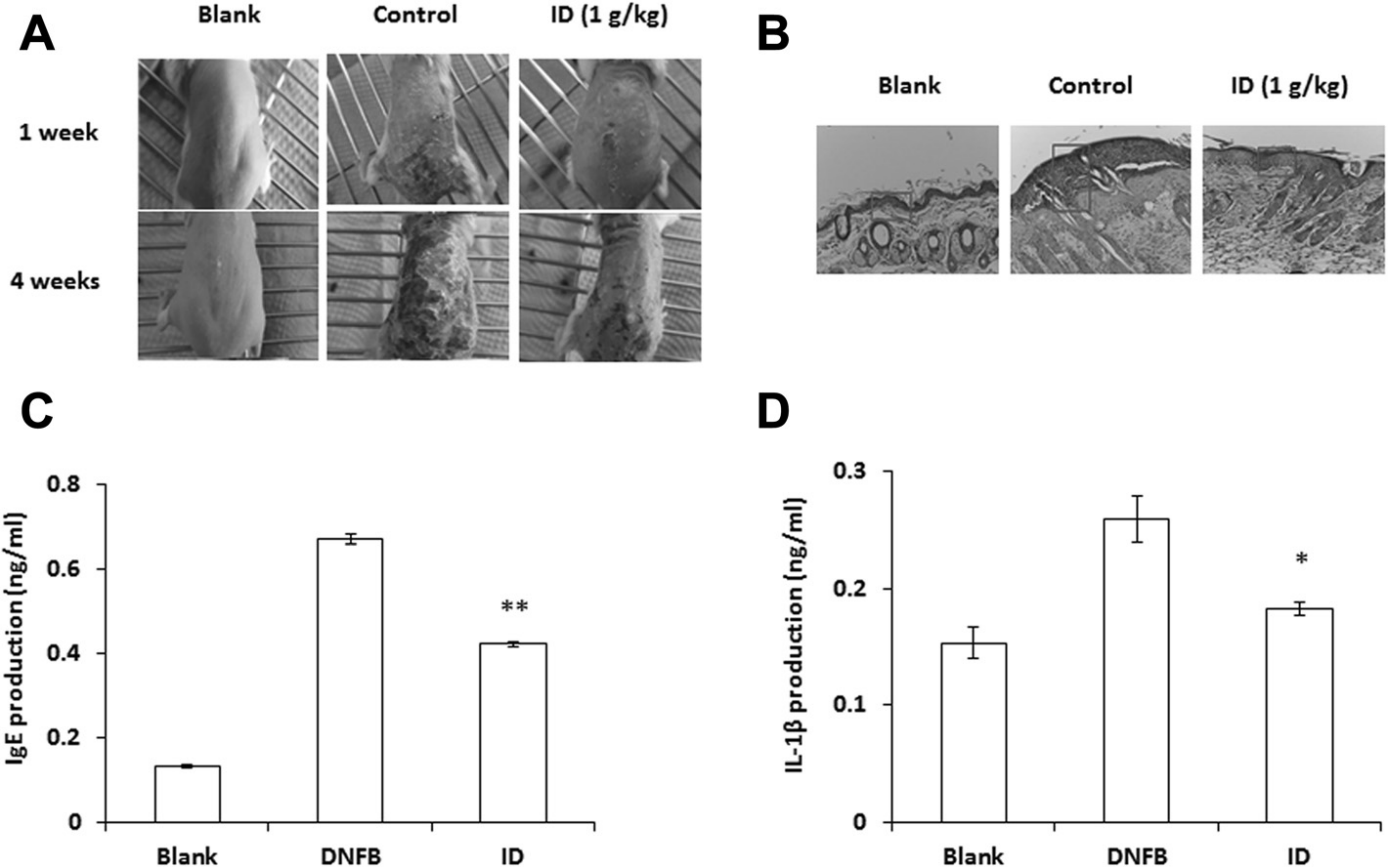
Effects of ID on DNFB-induced dermatitis and serum IgE and IL-1β levels. BALB/c mice (n = 7) were sensitized with 100 μl of 0.15 % DNFB in an acetone-olive oil combination (3: 1) or a vehicle (acetone/olive oil = 3: 1) applied to the dorsal skin twice each week for a total period of six weeks. ID (1 g/kg) was orally administrated to DNFB-treated mice for six weeks. (**a**) Comparison of DNFB-induced dermatitis in BALB/c mice after oral administration of ID. (**b**) H&E stained tissue sections. Skin tissues were fixed with 10 % formaldehyde embedded in paraffin. (**c** and **d**) Blood samples were collected from the mice, and the levels of serum IgE (**c**) and IL-1β (**d**) in the indicated groups were measured with the ELISA method. Values represent the mean ± SD of independent experiments. . *p < 0.05, **p < 0.01

The cutaneous application of DNFB was repeated to induce AD-like skin lesion in BALB/c mice. As shown in Fig. 1a, AD-like skin lesions were recovered in the group administrated ID as compared to the DNFB-treated group (control). The dermatitis skins were stained with hematoxylin and eosin (H&E) and evaluated by light microscopy. Marked accumulation of immune cells was observed in the control group, whereas the ID treatment inhibited hyperplasia (Fig. 1b). To evaluate the effects of ID on the inflammatory mediator production, blood samples were collected and analyzed using the ELISA method. Serum IgE and IL-1β levels were reduced by ID administration (0.4213 ± 0.02407 and 0.1956 ± 0.04152 ng/ml) compared to the control group (0.6677 ± 0.02048 and 0.2633 ± 0.00392 ng/ml) (Fig. 1c and d). These results indicated that the ID administration reduced dermal hyperplasia through the inhibition of inflammatory cell infiltration and inflammatory mediator production.

### Effect of ID on the phosphorylation of MAPKs and transcription factors in DNFB-induced skin lesions

**Fig. 2:**
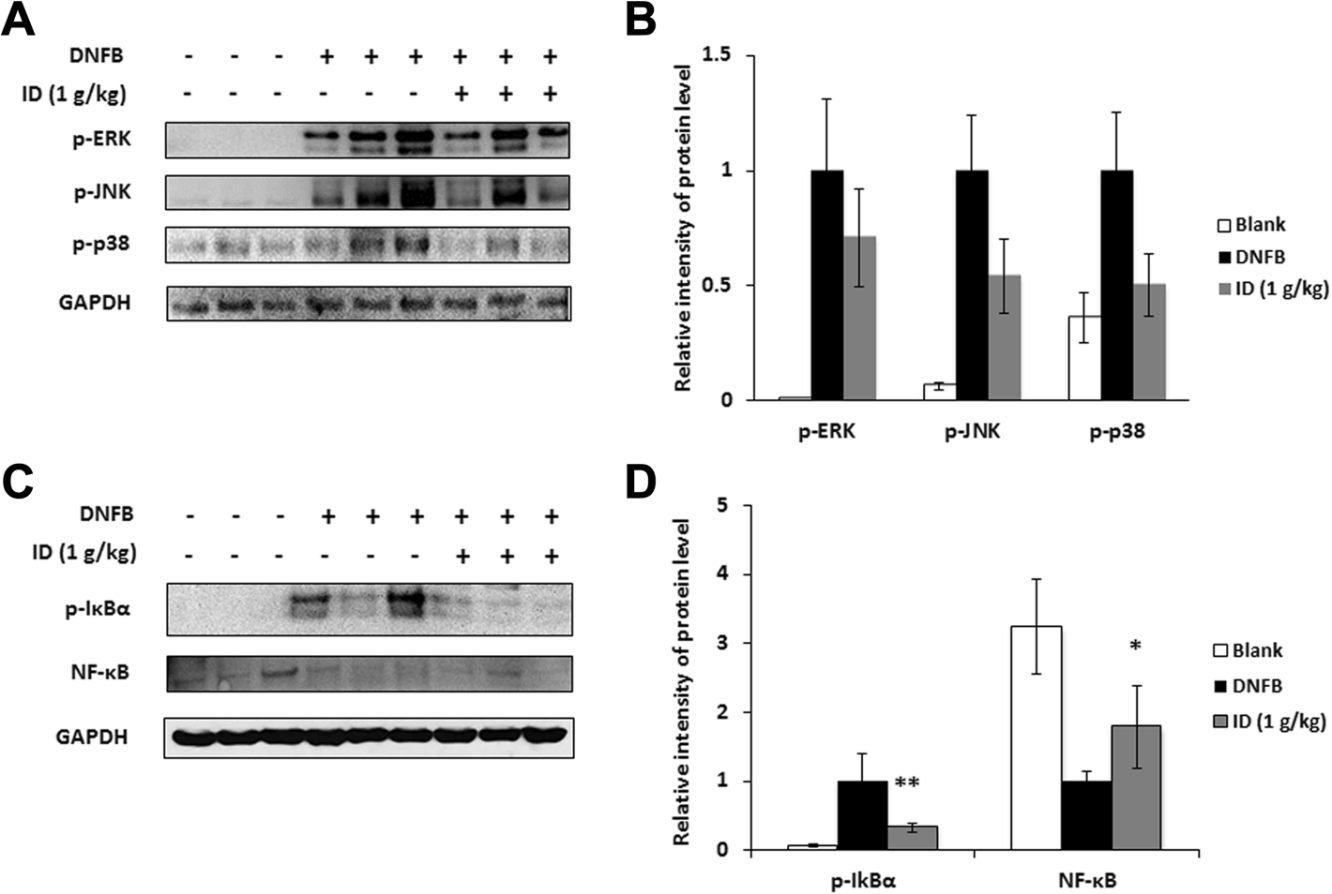
Effects of ID on activation of MAPKs and NF-κB in skin lesions. Protein was isolated from normal or DNFB-induced dermatitis dorsal skin. (**a**) Phosphorylation levels of ERK, JNK, and p38 was assayed by western blot analysis. (**b**) Relative levels of p-ERK, p-JNK, and p-p38 were calculated using an image analyzer. Values represent the mean ± SD of independent experiments. (**c**) The phosphorylation of IκBα and the protein expressions of NF-κB were assayed by western blot analysis. (**d**) The relative levels of p-IκBα and NF-κB were calculated using an image analyzer. Values represent the mean ± SD of independent experiments. *p < 0.05, **p < 0.01

The activation of MAPKs induces the production of proinflammatory cytokines and is highly activated in allergic reactions, including dermatitis [25]. Therefore, we evaluated the effect of ID on the phosphorylation of MAPKs. An ID treatment suppressed the phosphorylation of ERK, JNK, and p38 in dorsal skin compared to the control group (Fig. 2a). The relative expression levels of the MAPKs are represented in Fig. 2b. Because NF-κB is a pivotal transcription factor of inflammatory and immune reactions [22], the effect of ID on the activation of NF-κB was investigated. As shown in Fig. 2c, ID inhibited the phosphorylation of IκBα and NF-κB translocation into the nucleus. The relative expression levels of transcription factors are represented in Fig. 2d. These results suggest that ID inhibits the activation of MAPKs and NF-κB in AD-like skin lesions.

### Effect of ID on compound 48/80-induced anaphylactic shock

The inhibitory effect of ID on anaphylactic shock produced by compound 48/80 was evaluated using BALB/c mice. An oral administration of ID (0.01 - 1 g/kg) reduced the mortality rate induced by compound 48/80 in a dose-dependent manner (Table 2).

**Table 2:** Effect of ID on the compound 48/80-induced systemic anaphylactic reaction

ID (g/kg)^a^	Compound 48/80 (8 mg/kg)^b^	Mortality (%)^c^
None (saline)	+	100
0.01	+	80
0.1	+	73.3
1	+	46.6
1	−	0

^a^The groups of mice (*n* = 5) were orally administrated with saline or ID given at various doses 1 h before the compound 48/80 injection

^b^The compound 48/80 solution was intraperitoneally injected to the groups of mice

^c^Mortality (%) was presented as the ‘Number of dead mice × 100/Total number of experimental mice’. This result was represented by average results from three independent experiments

### Effects of ID and caffeic acid on the expression of chemokines in HaCaT cells

**Fig. 3:**
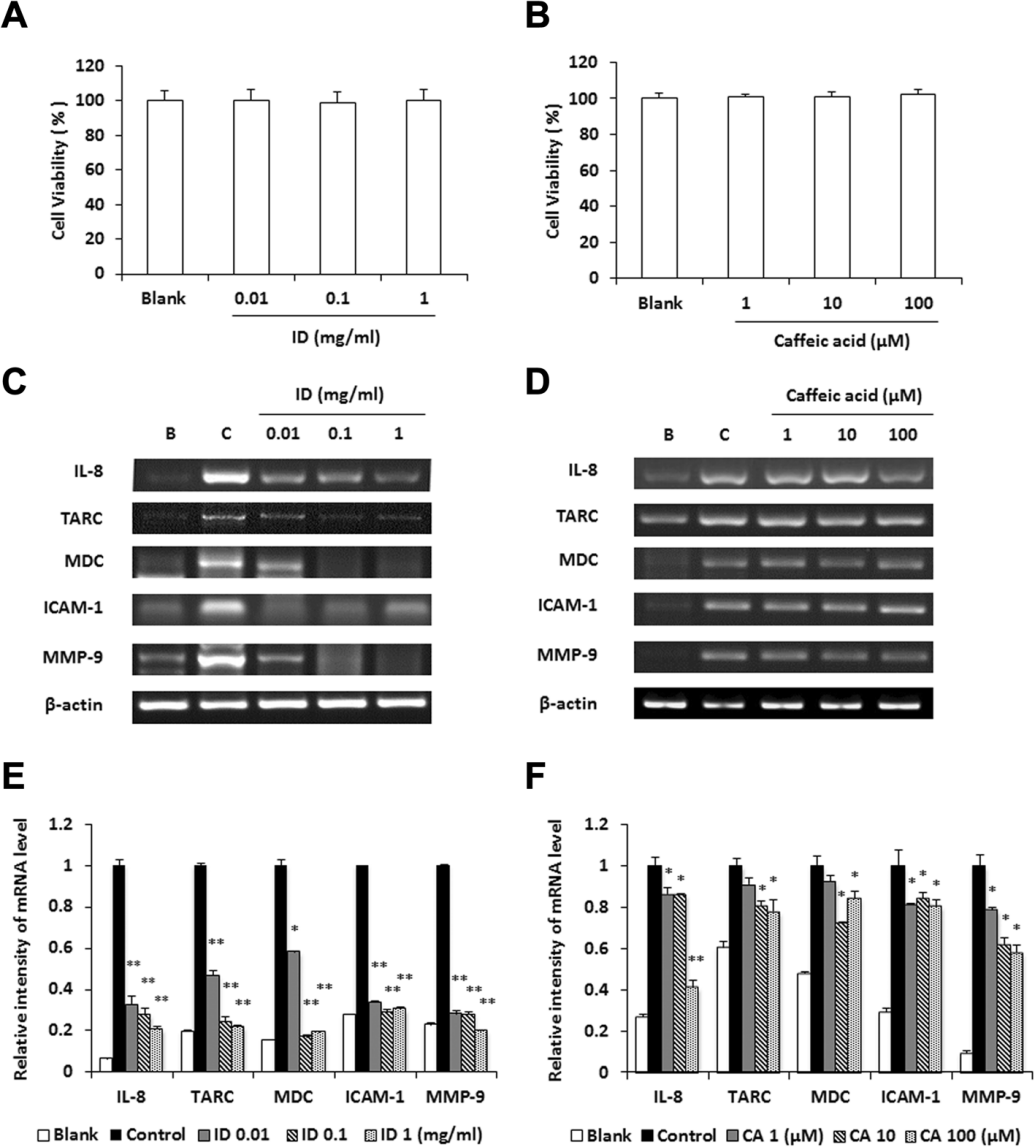
Effects of ID and caffeic acid on the expression levels of inflammatory mediators in HaCaT cells. (**a** and **b**) HaCaT cells (2 × 10^5^ cells/well) were treated with ID (0.01 - 1 mg/ml) or caffeic acid (1–100 μM) for 12 h and the degree of cell viability was analyzed by an MTT assay. (**c** and **d**) HaCaT cells (2 × 10^5^ cells/well) were pre-treated with ID or caffeic acid for 30 min and were stimulated with TNF-α/IFN-γ for 18 h. The levels of cytokine were assayed by a RT-PCR analysis. (**e** and **f**) Relative levels of IL-8, TARC, MDC, ICAM-1, and MMP-9 were calculated using an image analyzer. Values represent the mean ± SD of three independent experiments. CA, caffeic acid. *p < 0.05, **p < 0.01

Epidermal keratinocytes play multiple roles in the immune reactions associated with allergic contact dermatitis and atopic dermatitis skin diseases [12]. Therefore, the effect of ID on the degree of cell viability was first examined using an MTT assay. As shown in Fig. 3a, treatment with 0.01 - 1 mg/ml ID did not show cytotoxicity in HaCaT cells. To determine whether ID inhibits the production of TNF-α/IFN-γ-induced chemokines, we carried out a RT-PCR analysis and ELISA tests. The expression levels of chemokines (IL-8, TARC, and MDC), ICAM-1, and MMP-9 were suppressed by the ID treatment (Fig. 3c). The relative mRNA expression levels are represented in Fig. 3e. In previous research, we found through an HPLC analysis that caffeic acid was a major compound of ID [8]. Thus, we investigated the effect of caffeic acid on the production of cytokines and chemokines in HaCaT cells. Concentrations of caffeic acid were determined using MTT assay (Fig. 3b). Caffeic acid decreased chemokines including IL-8, TARC and MDC as well as ICAM-1 and MMP-9 expressions were decreased by the caffeic acid treatment (Fig. 3d). The relative mRNA expression levels are represented in Fig. 3f.

### Effects of ID on the production of proinflammatory cytokines and the activation of MAPKs in HMC-1 cells

**Fig. 4:**
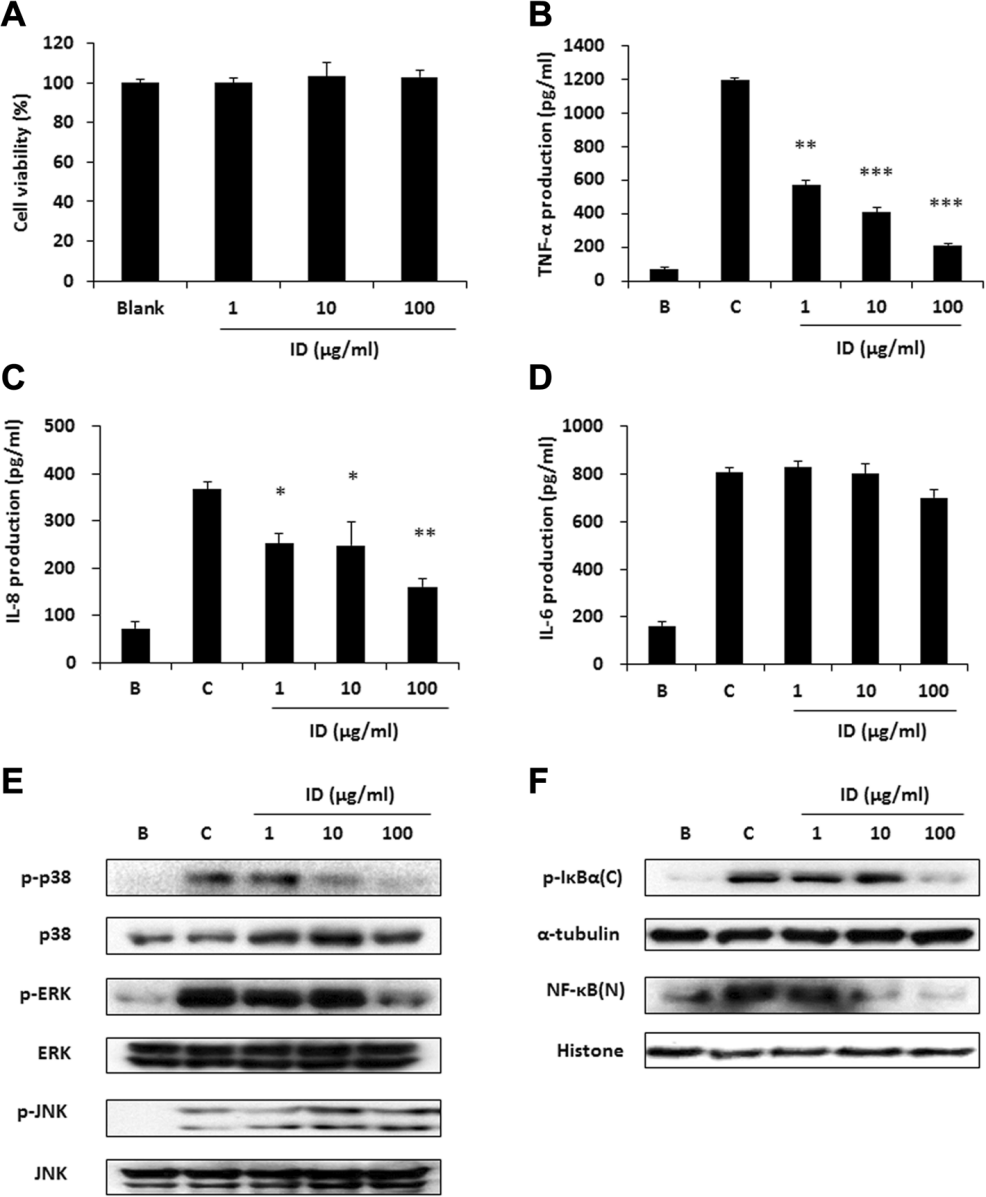
Effects of ID on the production of proinflammatory cytokines and the activation of MAPKs and NF-κB in HMC-1 cells. (**a**) HMC-1 cells (1 × 10^4^ cells/well) were treated with ID (0.01 - 1 mg/ml) for 24 h and the cell viability was analyzed with MTT assays. (**b**-**d**) The production levels of TNF-α (B), IL-8 (**c**), and IL-6 (**d**) were measured using the ELISA method. HMC-1 cells (2 × 10^5^ cells/well) were pre-treated with ID for 1 h and were stimulated with PMA (50 nM) + A23187 (1 μM) for 8 h. Values represent the mean ± SD of three independent experiments. *p < 0.05, **p < 0.01, ***p < 0.001. (**e** and **f**) Phosphorylation of p38, ERK and JNK (**e**) and the phosphorylation of IκBα and translocation of NF-κB (**f**) were assayed by western blot analysis. HMC-1 cells (1 × 10^6^ cells/well) were incubated with various concentrations of ID and then stimulated with PMA (50 nM) + A23187 (1 μM) for 30 min (**e**) or 2 h (**f**). C, cytosol extracts; N, nuclear extracts

Mast cells play a role in allergic reactions through the release of a number of mediators and cytokines [29]. We determined whether ID regulates the levels of proinflammatory cytokines in HMC-1 cells. First, we checked the cytotoxicity of ID on HMC-1 cells using an MTT assay (Fig. 4a). In the activated HMC-1 cells, ID significantly suppressed the production of TNF-α and IL-8, but not IL-6 (Fig. 4b-d). Moreover, we investigated the regulatory effects of ID on the activation of MAPKs. ID decreased the PMA + A23187-induced phosphorylation of p38 and ERK while the results for JNK showed no changes (Fig. 4e). ID also inhibited the phosphorylation of IκBα and the nuclear localization of NF-κB (Fig. 4f). These results suggest that ID suppresses the activation of NF-κB via p38 and ERK phosphorylation.

### Effects of caffeic acid on the production of proinflammatory cytokines and the activation of MAPKs in HMC-1 cells

**Fig. 5:**
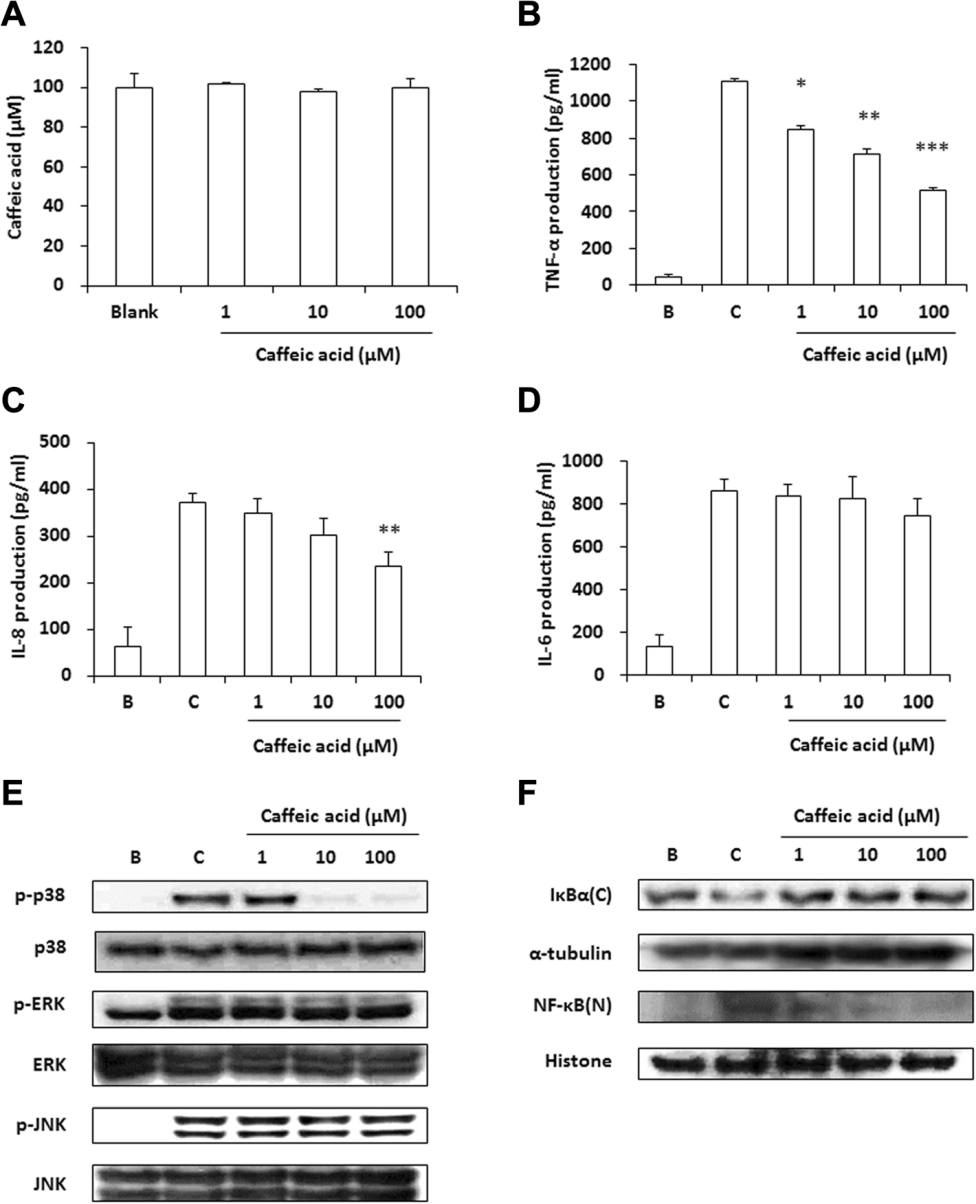
Effects of caffeic acid on the production of proinflammatory cytokines and the activation of MAPKs and NF-κB in HMC-1 cells. (**a**) HMC-1 cells (1 × 10^4^ cells/well) were treated with caffeic acid (1–100 μM) for 24 h and the degree of cell viability was analyzed with MTT assays. (**b**-**d**) The production levels of TNF-α (**b**), IL-8 (**c**), and IL-6 (**d**) were measured using the ELISA method. HMC-1 cells (2 × 10^5^ cells/well) were pre-treated with caffeic acid for 1 h and were stimulated with PMA (50 nM) + A23187 (1 μM) for 8 h. Values represent the mean ± SD of three independent experiments. *p < 0.05, **p < 0.01, ***p < 0.001. (**e** and **f**) Phosphorylation of p38, ERK and JNK (**e**) and the degradation of IκBα and translocation of NF-κB (**f**) were assayed by western blot analysis. HMC-1 cells (1 × 10^6^ cells/well) were incubated with various concentrations of caffeic acid and then stimulated with PMA (50 nM) + A23187 (1 μM) for 30 min (**e**) or 2 h (**f**). C, cytosol extracts; N, nuclear extracts

To investigate whether caffeic acid inhibits the production of cytokines, the TNF-α, IL-8, and IL-6 levels were measured by the ELISA method. The concentration of caffeic acid in HMC-1 cells was determined by an MTT assay (Fig. 5a). Caffeic acid significantly reduced the levels of TNF-α and IL-8, but not IL-6 (Fig. 5b-d). We examined the regulatory effects of caffeic acid on the phosphorylation of MAPKs (p38, ERK, and JNK). Caffeic acid decreased the PMA + A23187-induced phosphorylation of p38, while the results for ERK and JNK showed no changes (Fig. 5e). Additionally, caffeic acid inhibited the degradation of IκBα and the nuclear localization of NF-κB (Fig. 5f). These results suggest that caffeic acid suppresses the activation of NF-κB via the inhibition of p38 phosphorylation.

## Discussion

AD is a chronic inflammatory skin disease associated with cutaneous hyperreactivity to environmental triggers and skin inflammatory responses driven by cytokines expression. Furthermore, the incidence of AD has continued to increase over the past several decades [17]. Common treatments for AD are steroid therapy and immunosuppressive drugs, but these show side-effects, especially with continuous application [30]. Therefore, researchers have attempted to find a new drug in complementary and alternative medicines, such as herbal medicines [31, 32]. In this study, we demonstrated the regulatory effect of ID on allergic reactions *in vivo* and *in vitro*.

Haptens such as DNFB are used to induce a model of murine-contact hypersensitivity (MCH). It has been reported that MCH can generate AD-like skin lesions and immune responses with Th1-and/or Th2-type inflammation under certain conditions [33]. Dermatitis is characterized as a potent form of skin inflammation associated with an elevated level of IgE and the activation of IL-1β [34–36]. In this study, we mainly focused on the regulation of ID on proinflammatory cytokines but not Th2 cytokines, since it has been reported that the inhibitory effect of ID on DSS-induced colitis is due to the reduction of proinflammatory cytokines including TNF-α and IL-6 in previous study [8]. Therefore, we repeated the application of DNFB to induce AD-like lesions in BALB/c mice and investigated an effect of ID treatment. ID significantly reduced DNFB-induced dermatitis and decreased the serum levels of IgE and the IL-1β via the inhibition of p38, ERK, and JNK phosphorylation as well as the activation of NF-κB in AD-like skin lesions (Fig. 1 and 2). Compound 48/80, one of the most potent secretagogues, increases the permeability of the lipid bilayer membrane by causing a perturbation in the membrane upon mast cell degranulation [37]. ID administration also inhibited compound 48/80-induced anaphylactic shock in dose-dependent manner (Table 2). These results indicate that ID has a potent effect on anti-allergic responses in mice model.

HaCaT cells, a spontaneously transformed human keratinocyte cell line, are commonly used in pharmacological studies of skin diseases such as dermatitis, as it produces proinflammatory cytokines and chemokines upon various types of stimulation. HaCaT cells develop skin-associated lymphoid tissue by increasing the levels of surface antigens and interacting with other immunocytes [38]. TARC and MDC, typical Th2 chemokines, are both induced in HaCaT cells upon stimulation with IFN-γ and TNF-α [39]. As the chemokine, IL-8 plays a role in the activation of inflammatory effector cells such as neutrophils, T-lymphocytes, and eosinophils [40]. In this study, ID inhibited the mRNA expression of chemokines (IL-8, TARC, and MDC), and the adhesion molecule (ICAM-1), and MMP-9. Caffeic acid also reduced the expressions of IL-8 and TARC in TNF-α/IFN-γ-stimulated HaCaT cells (Fig. 3).

Mast cells play an important role in both adaptive and innate immunity and show increases in immunological skin diseases, including dermatitis [41]. The HMC-1 cell line was established from leukemia patients; it expresses characteristic markers for mast cells [42]. In response to different types of stimulation, mast cells release an array of cytokines, especially TNF-α, IL-6, and IL-8, with the potential to cause inflammation [18]. Therefore, the inhibition of cytokine secretion can be a useful therapeutic strategy for allergic inflammatory diseases such as AD. In this study, we demonstrated that ID and caffeic acid decreased the production of TNF-α and IL-8 in PMA + A23187-stimulated HMC-1 cells (Fig. 4 and 5). The expressions of proinflammatory cytokines such as TNF-α, IL-6, and IL-8 are associated with the activation of MAPKs and transcription factor NF-κB [25, 26]. To elucidate the mechanisms of the ID-mediated inhibition of proinflammatory cytokine production, we examined the regulatory effect of ID on the intracellular signaling molecules involved in the PMA + A23187-induced signaling pathways. ID decreases the nuclear translocation of NF-κB via the p38 and ERK, whereas caffeic acid regulates the phosphorylation of p38 but not ERK or JNK (Fig. 4 and 5). These results demonstrate that ID and caffeic acid suppress the production of proinflammatory cytokines through the phosphorylation of MAPKs and the activation of the NF-κB pathway. In contrast, we were unable to find the inhibitory effect of ID and caffeic acid on IL-6 production. Other studies demonstrated that IL-6 production is regulated by JNK activation in renal epithelial cells and PMA-stimulated Jurkat T cells [43, 44]. Since the drugs might not affect JNK activation, consequently both drugs did not decrease the IL-6 production.

In a previous study, we found that ID contained approximately 4 mg/g of caffeic acid (3,4-dihydroxy cinamic acid) [8]. Approximately 20 types of sesquiterpene and other compounds have been isolated from ID. It has been reported that its main compounds are luteolin, luteolin 7-O-glucuronide, caffeic acid, chlorogenic acid, and guaiane sesquiterpene lactones [45, 46]. Among these main compounds, inhibitory effects of luteolin, chlorogenic acid, and tectroside on allergic inflammation had already been reported [9, 47–49]. Thus, we focused on ID and caffeic acid and demonstrated both drugs’ anti-allergic inflammatory effect on HaCaT and HMC-1 cells. Caffeic acid was effective for reducing inflammatory responses with regard to TPA-induced ear thickness, and TPA/calcium ionophore or TNF-α-stimulated HaCaT cells [50, 51]. Therefore, caffeic acid may be partly responsible for the anti-allergic effect of ID. Indeed, we showed in present study that caffeic acid reduced the expressions of IL-8 and TARC as well as p38/NF-κB pathway in TNF-α/IFN-γ-stimulated HaCaT cells and HMC-1 cells.

In this study, ID decreased the production of proinflammatory cytokines including TNF-α and IL-8 in mast cells and inhibited TNF-α/IFN-γ-induced expression of chemokines such as IL-8, TARC and MDC in HaCaT cells. We showed that ID also reduced the expression of MMP-9, which is induced by histamine in keratinocyte [52]. Yi et al. [10] reported that ID inhibited the histamine release in mast cells. Therefore we suggest that reduced MMP-9 expression in keratinocytes by ID may be due to inhibition of ID on histamine release. These responses might also be occurred via blockage of the p38 and ERK/NF-κB pathway in mast cells and keratinocytes by ID treatment. In addition, MAPKs/NF-κB activation was inhibited by ID administration in AD-like skin lesion mice model. Consequently, ID water extract can improve the allergic inflammatory diseases through inhibition of proinflammatory cytokines, chemokines, and MMP-9 by regulation of MAPKs/NF-κB pathway in mast cells and keratinocytes.

## Conclusions

Our data suggest that ID can be used in the treatment of allergic skin diseases, including AD. ID showed anti-inflammatory and anti-allergic activities by suppressing skin dermatitis in DNFB-sensitized mice and compound 48/80-induced systemic anaphylaxis through the inhibition of serum IgE and IL-1β release. In addition, ID inhibited the TNF-α/IFN-γ-induced expression of chemokines (IL-8, TARC, and MDC), ICAM-1, and MMP-9 in keratinocytes. Moreover, ID reduced proinflammatory cytokines (TNF-α and IL-8) via the blockage of p38/ERK phosphorylation and NF-κB activation in mast cells. These results lend insight into the pharmacological actions of ID as a potential therapy for allergic inflammatory diseases such as dermatitis.
